# Increase in endogenous glucose production with SGLT2 inhibition is attenuated in individuals who underwent kidney transplantation and bilateral native nephrectomy

**DOI:** 10.1007/s00125-020-05254-w

**Published:** 2020-08-22

**Authors:** Giuseppe Daniele, Carolina Solis-Herrera, Angela Dardano, Andrea Mari, Andrea Tura, Laura Giusti, Jancy J. Kurumthodathu, Beatrice Campi, Alessandro Saba, Anna Maria Bianchi, Carla Tregnaghi, Maria Francesca Egidi, Muhammad Abdul-Ghani, Ralph DeFronzo, Stefano Del Prato

**Affiliations:** 1 Department of Clinical and Experimental Medicine, Section of Metabolic Diseases and Diabetes, University of Pisa, Via Paradisa 2, 56124 Pisa, Italy; 2 Division of Diabetes, University of Texas Health Science Center at San Antonio, San Antonio, TX USA; 3 Metabolic Unit, CNR Institute of Neuroscience, Padova, Italy

**Keywords:** Dapagliflozin, Endogenous glucose production, Glucosuria

## Abstract

**Aims/hypothesis:**

The glucosuria induced by sodium–glucose cotransporter 2 (SGLT2) inhibition stimulates endogenous (hepatic) glucose production (EGP), blunting the decline in HbA_1c_. We hypothesised that, in response to glucosuria, a renal signal is generated and stimulates EGP. To examine the effect of acute administration of SGLT2 inhibitors on EGP, we studied non-diabetic individuals who had undergone renal transplant with and without removal of native kidneys.

**Methods:**

This was a parallel, randomised, double-blind, placebo-controlled, single-centre study, designed to evaluate the effect of a single dose of dapagliflozin or placebo on EGP determined by stable-tracer technique. We recruited non-diabetic individuals who were 30–65 years old, with a BMI of 25–35 kg/m^2^ and stable body weight (±2 kg) over the preceding 3 months, and HbA_1c_ <42 mmol/mol (6.0%). Participants had undergone renal transplant with and without removal of native kidneys and were on a stable dose of immunosuppressive medications. Participants received a single dose of dapagliflozin 10 mg or placebo on two separate days, at a 5- to 14-day interval, according to randomisation performed by our hospital pharmacy, which provided dapagliflozin and matching placebo, packaged in bulk bottles that were sequentially numbered. Both participants and investigators were blinded to group assignment.

**Results:**

Twenty non-diabetic renal transplant patients (ten with residual native kidneys, ten with bilateral nephrectomy) participated in the study. Dapagliflozin induced greater glucosuria in individuals with residual native kidneys vs nephrectomised individuals (8.6 ± 1.1 vs 5.5 ± 0.5 g/6 h; *p* = 0.02; data not shown). During the 6 h study period, plasma glucose decreased only slightly and similarly in both groups, with no difference compared with placebo (data not shown). Following administration of placebo, there was a progressive time-related decline in EGP that was similar in both nephrectomised individuals and individuals with residual native kidneys. Following dapagliflozin administration, EGP declined in both groups, but the differences between the decrement in EGP with dapagliflozin and placebo in the group with bilateral nephrectomy (Δ = 1.11 ± 0.72 μmol min^−1^ kg^−1^) was significantly lower (*p* = 0.03) than in the residual native kidney group (Δ = 2.56 ± 0.33 μmol min^−1^ kg^−1^). In the population treated with dapagliflozin, urinary glucose excretion was correlated with EGP (*r* = 0.34, *p* < 0.05). Plasma insulin, C-peptide, glucagon, prehepatic insulin:glucagon ratio, lactate, alanine and pyruvate concentrations were similar following placebo and dapagliflozin treatment. β-Hydroxybutyrate increased with dapagliflozin treatment in the residual native kidney group, while a small increase was observed only at 360 min in the nephrectomy group. Plasma adrenaline (epinephrine) did not change after dapagliflozin and placebo treatment in either group. Following dapagliflozin administration, plasma noradrenaline (norepinephrine) increased slightly in the residual native kidney group and decreased in the nephrectomy group.

**Conclusions/interpretation:**

In nephrectomised individuals, the hepatic compensatory response to acute SGLT2 inhibitor-induced glucosuria was attenuated, as compared with individuals with residual native kidneys, suggesting that SGLT2 inhibitor-mediated stimulation of hepatic glucose production via efferent renal nerves occurs in an attempt to compensate for the urinary glucose loss (i.e. a renal–hepatic axis).

**Trial registration:**

ClinicalTrials.gov NCT03168295

**Funding:**

This protocol was supported by Qatar National Research Fund (QNRF) Award No. NPRP 8-311-3-062 and NIH grant DK024092-38.

Graphical abstract
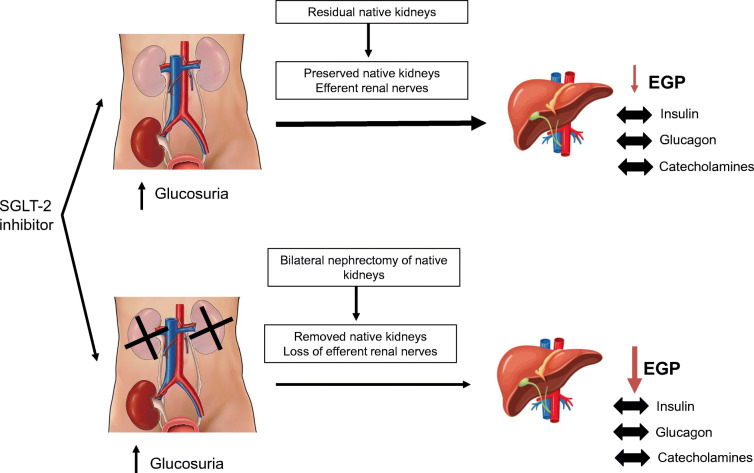

**Electronic supplementary material:**

The online version of this article (10.1007/s00125-020-05254-w) contains supplementary material, which is available to authorized users.

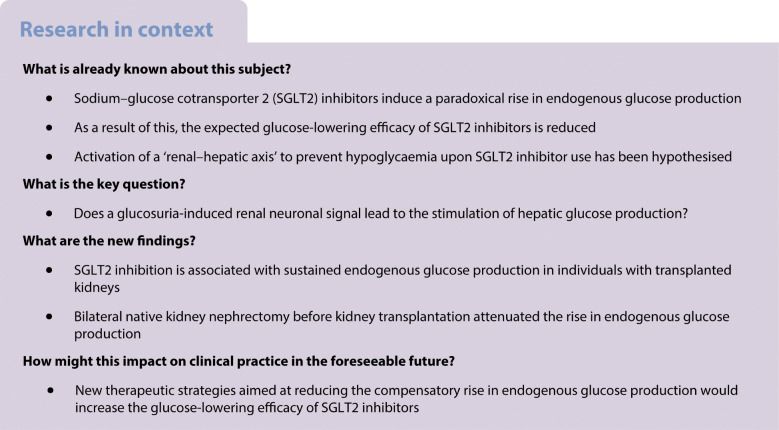



## Introduction

Sodium–glucose cotransporter 2 (SGLT2) inhibitors reduce plasma glucose concentration by blocking renal glucose–sodium cotransport and inducing glucosuria. The expected reduction in plasma glucose concentration is partially offset by a concomitant increase in endogenous glucose production (EGP) [1, 2]. When non-diabetic individuals are given the SGLT2 inhibitor empagliflozin, urinary glucose excretion increases promptly, yet the fasting plasma glucose concentration remains unchanged [3]. These results suggest that the induction of glucosuria generates a signal that increases EGP to precisely match the amount of glucose appearing in the urine. The increase in plasma glucagon concentration and concomitant reduction in plasma insulin concentration cannot explain this ‘paradoxical’ and rapid increase in EGP [1, 2]. Because changes in plasma glucagon and insulin concentrations cannot explain the increase in EGP [1, 2], we hypothesised that a renal neuronal signal is generated in response to glucosuria and leads to the stimulation of hepatic glucose production. Consistent with this hypothesis, renal sympathetic denervation for the treatment of resistant hypertension has been shown to improve glucose tolerance [4, 5]. Kidney transplantation offers another model of kidney denervation. We have recently assessed the effect of administration of 10 mg dapagliflozin or placebo after an overnight fasting in 14 individuals (six with type 2 diabetes and eight without diabetes) who received a renal transplant [6]. In both diabetic and non-diabetic transplanted individuals, SGLT2 inhibitor-mediated stimulation of EGP was still fully apparent. The degree of renal denervation in these individuals is, however, difficult to evaluate due to the persistence of the native kidney and its related innervation. The objectives of the current study were to examine whether a neuronal loop may exist between the kidney and the liver, accounting for sustained hepatic glucose production after administration of an SGLT2 inhibitor.

## Methods

### Study population

Non-diabetic renal transplant recipients with eGFR >60 ml min^−1^ [1.73 m]^−2^ (using the Chronic Kidney Disease Epidemiology Collaboration [CKD-EPI] formula) were recruited from the Nephrology Unit of the Department of Clinical and Experimental Medicine at the University of Pisa, Italy. All participants underwent renal transplantation because of autosomal dominant polycystic renal disease. Inclusion criteria were: age 30–65 years; BMI 25–35 kg/m^2^ with stable body weight (±2 kg) over the preceding 3 months; and HbA_1c_ <42 mmol/mol (6.0%). Ten renal transplant patients with both residual native kidneys (*n* = 10) and ten renal transplant patients who underwent bilateral nephrectomy prior to transplantation comprised the study population. Participants were on a stable dose of combinations of the following immunosuppressive medications: mycophenolate mofetil, tacrolimus, everolimus, sirolimus; prednisone <7.5 mg/day; or ciclosporin. Individuals on >7.5 mg/day prednisone, β-blockers or any other medication known to affect sympathetic/parasympathetic activity or glucose metabolism were excluded. Participants with New York Heart Association (NYHA) class III–IV heart failure or BP >140/90 mmHg were excluded.

The Institutional Review Board of the University of Pisa and the Italian Medicines Agency (AIFA) approved the study in accordance with principles described in the Declaration of Helsinki. All participants gave written informed consent before participating in the study. The study was registered at ClinicalTrials.gov (registration no. NCT03168295).

### Study design

This was a randomised, double-blind, placebo-controlled, single-centre study evaluating the effect of dapagliflozin on glucose metabolism in two groups of renal transplant participants: ten individuals with residual native kidneys and ten individuals who had undergone pre-transplant bilateral nephrectomy. At baseline, all participants had their medical history taken and underwent physical examination and routine laboratory tests to exclude major organ system disease other than polycystic kidney disease. Participants underwent two identical study procedures at a 5- to 14-day interval, with the administration of 10 mg dapagliflozin or placebo in randomised double-blind fashion. Dapagliflozin and matching placebo were packaged in bulk bottles and delivered to the hospital pharmacy, which managed the code list and supplied the study drug to the investigators on the study day using a binary randomisation code provided by www.random.org (accessed 1 October 2016). All procedures were performed after a 10 h overnight fast, at the Diabetes Section of the Department of Clinical and Experimental Medicine, University of Pisa. On admission, after voiding, a vein on the dorsum of the hand was cannulated retrogradely and the hand was placed in a heated box (60°C) for arterialised blood sampling. A second catheter was placed in the contralateral antecubital vein and a primed (22 μmol/kg), constant (0.22 μmol min^−1^ kg^−1^) infusion of [6,6-^2^H_2_]glucose was initiated at −120 min and maintained until the end of study (360 min). During the last 30 min of the equilibration period (from −30 min to 0 min) indirect calorimetry was performed using a ventilated hood system (Vmax 22 N; Sensor Medics, Yorba Linda, CA, USA). Arterialised blood samples were obtained at −120, −30, −20, −10 and 0 min for the measurement of plasma glucose, insulin, C-peptide, glucagon, adrenaline (epinephrine), noradrenaline (norepinephrine), lactate, alanine, β-hydroxybutyrate (βOHB), pyruvate concentrations and [6,6-^2^H_2_] glucose enrichment.

After the 2 h (−120 to 0 min) equilibration period and baseline blood and urine collection for measurement of glucose and nitrogen excretion, participants received dapagliflozin (10 mg) or placebo orally (at 0 min). Arterialised blood samples were drawn every 20 min for plasma glucose measurement and [6,6-^2^H_2_]glucose enrichment and every 60 min for measurement of insulin, C-peptide and glucagon. Adrenaline and noradrenaline were measured at 240, 340 and 360 min. Lactate, alanine, βOHB and pyruvate concentrations were measured at 180, 240 and 360 min. Indirect calorimetry was performed during the last 30 min of the study (330–360 min) and urine was collected from 0 min to −360 min for measurement of glucose and nitrogen excretion. Participants remained fasted until the conclusion of the experimental procedure during which they were allowed to have no more than 1 l of water. Participants were instructed not to change their dietary habits and energy expenditure during the entire study participation.

**Fig. 1:**
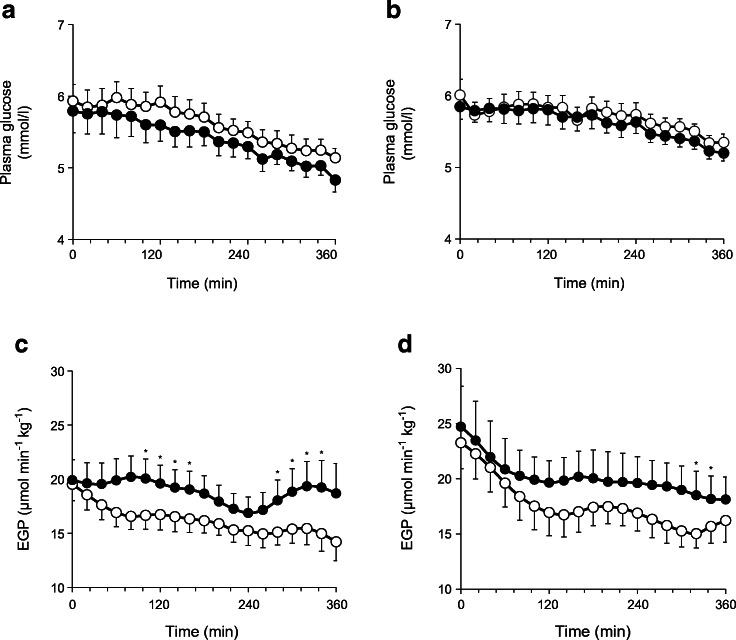
Plasma glucose (**a**, **b**) and EGP (**c**, **d**) following administration of placebo (white circles, *n* = 10) and dapagliflozin (black circles, *n* = 10) in individuals with residual native kidneys (**a**, **c**) and nephrectomised individuals (**b**, **d**). Data are presented as mean ± SEM. *p < 0.05 vs placebo

See electronic supplementary material (ESM) Fig. 1.

### Analytical techniques

Plasma glucose was measured by the glucose oxidase method (Analox GM9 Analyzer; Analox Instruments, London, UK). Plasma insulin, C-peptide (Pantec, Turin, Italy) and glucagon (DIAsource ImmunoAssays, Belgium) concentrations were measured by RIA. Plasma adrenaline and noradrenaline were measured by ELISA (DIASource ImmunoAssays). Plasma lactate, alanine, βOHB and pyruvate were measured by in-house automated spectrophotometric enzymatic methods on a Beckman UniCel DXC600 Synchron Analyser (Fullerton, CA, USA). Intra-assay and between-assay coefficients of variation were <1% and <5%, respectively, for all hormone and substrate measurements. Urinary nitrogen excretion was determined by chemiluminescence (Antek MultiTek Analyzer PAC, TX, USA). [6,6-^2^H_2_]glucose and [U-^13^C_6_]glucose (glucose-13C6), used as internal standards, were purchased from Cambridge Isotope Laboratories (Andover, MA, USA). Plasma isotope enrichment was measured on an AB Sciex API 4000 triple quadrupole mass spectrometer (Concord, ON, Canada), equipped with ESI Turbo-V source and coupled with Agilent 1290 Infinity UHPLC system (Santa Clara, CA, USA) working under gradient conditions.

### Calculations

Glucose fluxes were computed using a circulatory model and expressed per kg of body weight per min [7]. This model has been employed in prior human studies [8–10]. Briefly, a calibration curve is first created, which expresses the tracer:tracee ratio (TTR) (i.e. the [6,6-^2^H_2_]glucose:glucose peak ratio). From the calibration curve, the following equation was derived:

$$ \mathrm{TTR}=1.3074\times r-0.014675 $$

where *r* is the [6,6-^2^H_2_]glucose:glucose peak. From the TTR and the measured plasma glucose concentration, the tracer concentration can be obtained. Glucose clearance was then calculated based on tracer infusion rate and plasma tracer concentration. This was obtained by parameter estimation procedure through the model [7], with the fit of the tracer data. The estimated clearance was then used in the model, through mathematical deconvolution-like operation, to quantify EGP based on plasma glucose concentration. The rate of disappearance (*R*_d_) for glucose was calculated from the model-derived endogenous glucose concentration and glucose clearance rate. Tissue glucose utilisation was calculated as the difference between total body glucose *R*_d_ and urinary glucose excretion.

Energy expenditure and carbohydrate and lipid oxidation rates were determined according to established equations [11, 12]. Prehepatic insulin:glucagon molar concentration ratio was calculated as previously described [2].

### Statistical analysis

Based on prior studies [1, 2], we calculated that ten participants per group were sufficient to demonstrate a statistically significant difference in EGP between dapagliflozin- and placebo-treated individuals at *α* < 0.05 with 95% power. The effect of dapagliflozin on EGP and plasma hormone and metabolite concentrations was compared in nephrectomy vs residual native kidney groups by ANOVA. Differences from baseline were plotted against time in placebo-treated and dapagliflozin-treated patients and tested for significance using repeated measures ANOVA with time and treatment group as factors. Bonferroni test was used for post hoc testing. Differences in EGP and plasma hormone and metabolite concentrations (insulin, C-peptide, glucagon, adrenaline, noradrenaline, lactate, alanine, βOHB, pyruvate, glucose) between the dapagliflozin and placebo groups were compared using the Mann–Whitney *U* test.

## Results

The recruitment flow is shown in ESM Fig. 2. Of 50 participants who were screened, 25 were considered eligible and were randomised to the study intervention. Five people dropped out of the study: one nephrectomised individual withdrew consent prior to any drug administration as this individual moved to another town and another did not undergo the repeat study because of difficulty in inserting the i.v. catheter for blood withdrawal; among individuals with residual native kidneys, two withdrew consent prior to any drug administration for work-related reasons and one did not undergo the repeat study because of difficulty inserting the i.v. catheter for blood withdrawal. In total, ten nephrectomised individuals and ten individuals with residual native kidneys were randomised to dapagliflozin and placebo and completed both studies. As shown in Table 1, the two groups were well matched for baseline clinical, laboratory and anthropometric characteristics. All participants were taking a stable 5 mg prednisone dose per day for the past 3 months. No harms or unintended effects was recorded among patients completing the study.

**Table 1:** Main anthropometric and metabolic parameters of the study population

Characteristics	Residual native kidneys	Bilateral nephrectomy	*p* value
*N*	10	10	
Age (years)	52 ± 10	58.9 ± 9	0.21
Sex, female/male (*n*)	9/11	7/3	0.31
Transplant duration (years)	5.7 ± 0.5	8.5 ± 1.2	0.05
Weight (kg)	74.8 ± 4.5	74.2 ± 3.6	0.92
BMI (kg/m^2^)	25.5 ± 1.5	25.8 ± 1.5	0.88
Systolic BP (mmHg)	129 ± 6	132 ± 5	0.66
Diastolic BP (mmHg)	87 ± 4	80 ± 2	0.13
Fasting plasma glucose (mmol/l)	5.6 ± 0.2	5.5 ± 0.2	0.90
HbA_1c_ (mmol/mol)	40.1 ± 1.5	39.5 ± 2.0	0.89
HbA_1c_ (%)	5.8 ± 0.1	5.8 ± 0.1	
Plasma creatinine (μmol/l)	120 ± 7	109 ± 7	0.27
eGFR (ml min^−1^ [1.73 m]^−2^)	60.0 ± 4.7	63.7 ± 6.8	0.65
Total cholesterol (mmol/l)	4.9 ± 0.1	5.1 ± 0.4	0.54
LDL-cholesterol (mmol/l)	2.8 ± 0.12	2.6 ± 0.2	0.41
HDL-cholesterol (mmol/l)	1.3 ± 0.2	1.3 ± 0.1	0.68
Triacylglycerols (mmol/l)	1.4 ± 0.2	1.5 ± 0.2	0.79

Data are presented as mean ± SEM or *n*

### Plasma glucose concentration, urinary glucose excretion and glucose turnover

Dapagliflozin caused marked glucosuria in both groups, although this was higher in individuals with residual native kidneys as compared with the bilateral nephrectomy group (8.6 ± 1.1 vs 5.5 ± 0.5 g/6 h; *p* = 0.02). There was no detectable urinary glucose excretion after placebo administration. The fasting plasma glucose concentration was similar in both groups and decreased slightly over the 360 min study period (−0.88 ± 0.20 mmol/l in the bilateral nephrectomy group and −0.60 ± 0.10 mmol/l in the residual native kidney group), with no difference vs placebo (Fig. 1a, b).

Baseline EGP was slightly higher in the bilateral nephrectomy vs residual native kidney group after both dapagliflozin and placebo administration (Fig. 1c, d). After placebo administration, EGP declined progressively and similarly in both groups (Fig. 1c, d). Following dapagliflozin administration, the decline in EGP (120–360 min) was less marked in the residual native kidney group when compared with placebo administration (0.99 ± 0.11 vs 3.55 ± 0.44 μmol min^−1^ kg^−1^; *p* = 0.01; Table 2), showing persistence of EGP in the residual native kidney group as previously described [1]. In the group with bilateral nephrectomy who received dapagliflozin, the decline in EGP (120–360 min) was of marginal statistical significance compared with placebo (4.60 ± 1.33 vs 5.71 ± 0.61 μmol min^−1^ kg^−1^; *p* = 0.06) (Table 2).

**Table 2:** EGP before and after single-dose administration of dapagliflozin or placebo

Variable	Residual native kidneys (*n* = 10)		Bilateral nephrectomy (*n* = 10)	
	Placebo	Dapagliflozin	Placebo	Dapagliflozin
Baseline EGP (μmol min^−1^ kg^−1^)	19.48 ± 1.49	19.87 ± 1.88	23.25 ± 2.33	24.70 ± 3.66
Mean EGP from 120–360 min (μmol min^−1^ kg^−1^)	15.93 ± 1.05	18.88 ± 1.77	17.54 ± 1.72	20.10 ± 2.33
Difference between baseline and mean EGP (120–360 min)	3.55 ± 0.44	0.99 ± 0.11^§^	5.71 ± 0.61	4.60 ± 1.33^†^
Δ for dapagliflozin vs placebo		2.56 ± 0.33		1.11 ± 0.72^‡^

Data are presented as mean ± SEM

^§^*p* = 0.01 vs placebo within residual native kidney group; ^†^*p* = 0.06 vs placebo within bilateral nephrectomy group; ^‡^*p* = 0.03, residual native kidneys vs bilateral nephrectomy

Most importantly, the difference between the decrement in EGP between dapagliflozin and placebo in the group with bilateral nephrectomy (Δ = 1.11 ± 0.72 μmol min^−1^ kg^−1^) was significantly lower (*p* = 0.03) than in the residual native kidney group (Δ = 2.56 ± 0.33 μmol min^−1^ kg^−1^; Table 2). Thus, bilateral nephrectomy attenuated the ‘paradoxical’ stimulation of EGP by dapagliflozin.

**Fig. 2:**
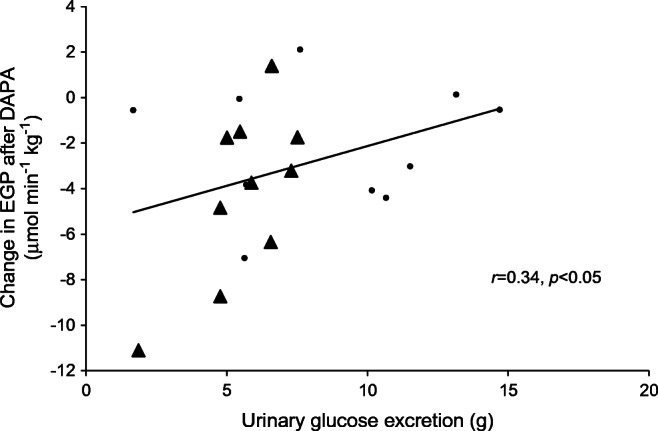
Pearson correlation between urinary glucose excretion and the change from baseline in EGP (120–360 min) following dapagliflozin administration. Circles, individuals with residual native kidneys; triangles, nephrectomised individuals. DAPA, dapagliflozin

**Fig. 3:**
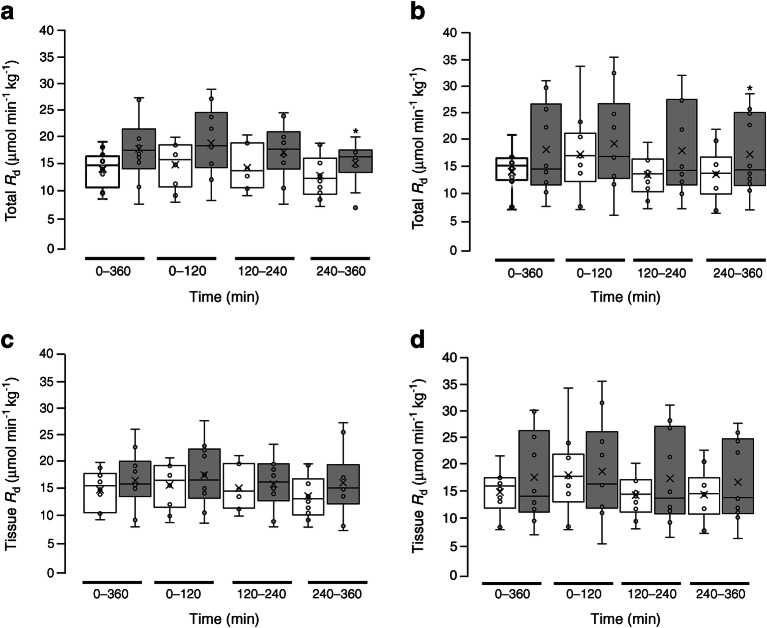
Total glucose *R*_d_ (**a**, **b**) and tissue glucose *R*_d_ (**c**, **d**) in individuals with residual native kidneys (**a**, **c**) and nephrectomised individuals (**b**, **d**). White bars, placebo; grey bars, dapagliflozin. Data are presented as box and whisker plots in which the central horizontal line is the median for each group, the boxes represent the first and third quartiles, and the whiskers represent the range of the data. *p < 0.05, dapagliflozin vs placebo at indicated timepoint

In the study population as a whole, following dapagliflozin administration, the amount of glucose excreted in the urine and the change in EGP from baseline were correlated (*r* = 0.34, *p* < 0.05) (Fig. 2). At baseline, the glucose *R*_d_ was slightly higher in the bilateral nephrectomy vs residual native kidney group (Fig. 3). Following placebo administration, the total glucose *R*_d_ declined slightly, but not significantly, in both groups along with the decline in plasma glucose concentration. After dapagliflozin administration, the total glucose *R*_d_ remained at the baseline level so that *R*_d_ was greater than after placebo administration (*p* < 0.01) during the last 2 h (240–360 min) of the study in both the nephrectomy group and the residual native kidney group. Tissue glucose *R*_d_ (i.e. total *R*_d_ minus urinary glucose excretion) was similar in both groups during all time periods (Fig. 3). There were no significant differences in the rates of oxidation of carbohydrate, lipid and protein, or in energy expenditure, between the nephrectomy group and residual native kidney group (ESM Table 1).

### Effect of dapagliflozin on plasma hormone and metabolite concentrations

**Fig. 4:**
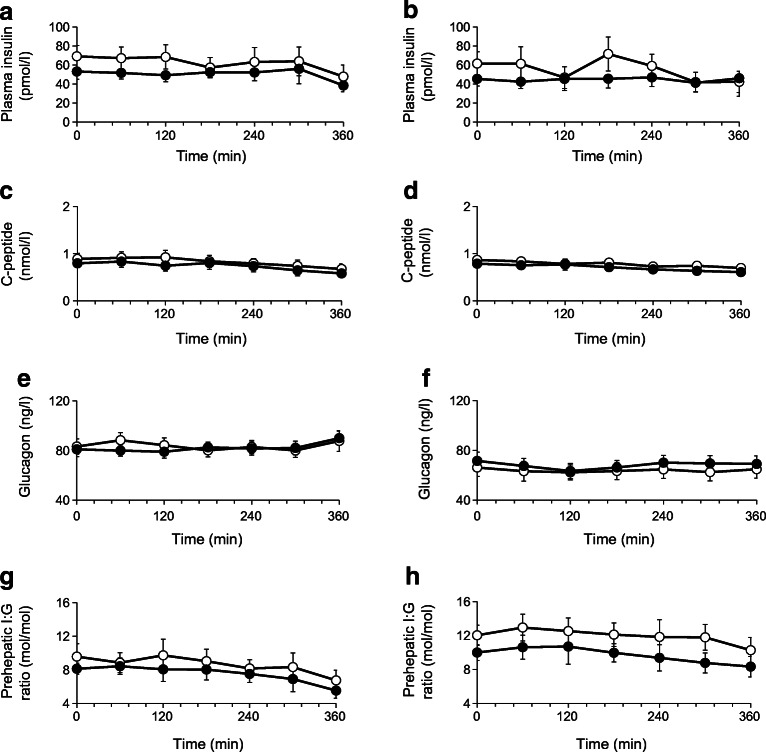
Time-related change in plasma insulin (**a**, **b**), C-peptide (**c**, **d**) and glucagon (**e**, **f**) concentrations, and prehepatic insulin:glucagon (I:G) ratio (**g**, **h**) following administration of placebo (white circles; *n* = 10) and dapagliflozin (black circles; *n* = 10) in individuals with residual native kidneys (**a**, **c**, **e**, **g**) and nephrectomised individuals (**b**, **d**, **f**, **h**). Data are presented as mean ± SEM

**Fig. 5:**
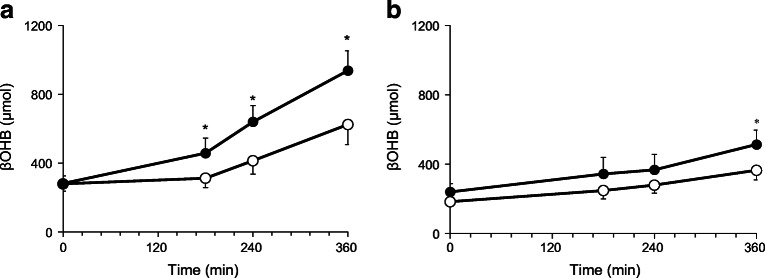
Plasma βOHB concentration following administration of placebo (white circles; *n* = 10) and dapagliflozin (black circles; *n* = 10) in individuals with residual native kidneys (**a**) and nephrectomised individuals (**b**). Data are presented as mean ± SEM. *p < 0.05, dapagliflozin vs placebo at indicated timepoint

The fasting plasma insulin, C-peptide and glucagon concentrations were not statistically different between the two groups (Fig. 4). Plasma insulin and C-peptide concentration showed a similar trend toward progressive reduction after administration of both dapagliflozin and placebo while plasma glucagon levels remained unchanged. There were no differences in the baseline and post-drug prehepatic insulin:glucagon ratio in either nephrectomised individuals or individuals with residual native kidneys following both dapagliflozin and placebo. Dapagliflozin administration was associated with a significant increase in plasma βOHB at all measured time points in individuals with residual native kidneys while a small increase in βOHB was observed only at 360 min in the bilateral nephrectomy group (Fig. 5). No significant changes in plasma lactate, pyruvate and alanine concentrations were observed after either dapagliflozin or placebo in either group (data not shown).

### Effect of dapagliflozin on catecholamines

**Fig. 6:**
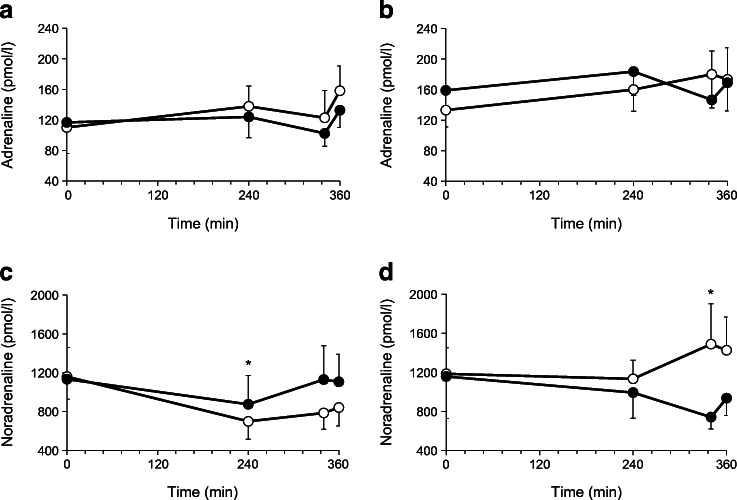
Plasma adrenaline (**a**, **b**) and noradrenaline (**c**, **d**) concentrations following administration of placebo (white circles; *n* = 10) and dapagliflozin (black circles; *n* = 10) in individuals with residual native kidneys (**a**, **c**) and bilateral nephrectomised individuals (**b**, **d**). Data are presented as mean ± SEM. *p < 0.05, dapagliflozin vs placebo at indicated timepoint

Plasma adrenaline levels were similar at baseline and did not change following dapagliflozin or placebo administration in nephrectomised individuals with residual native kidney (Fig. 6). At baseline, plasma noradrenaline concentration was similar under all conditions (Fig. 6). After dapagliflozin administration, when compared with placebo administration, plasma noradrenaline concentrations were slightly higher in individuals with residual native kidneys, while they were slightly lower in the bilateral nephrectomy group (Fig. 6).

## Discussion

In a recent study we showed that, in kidney transplanted individuals with and without type 2 diabetes, administration of a single dose of dapagliflozin was associated with a compensatory increase in EGP [6]. This is indicative of a minor role for kidney innervation in modulating the liver adaptative response. In that study, all transplanted individuals had their native kidneys in place. In the present study we went further in testing the hypothesis of a kidney–liver neuronal loop by comparing transplanted individuals with and without residual native kidneys. We found that, in the post-transplant group with both residual native kidneys, the stimulation of EGP by dapagliflozin-induced glucosuria was apparent, as evidenced by the failure of EGP to decline significantly when compared with the decline observed with the placebo (between-group differences in EGP = 1.45 ± 0.39 μmol min^−1^ kg^−1^; Table 2). This observation is consistent with previous data from individuals with type 2 diabetes treated with an SGLT2 inhibitor [1, 2], as well as from transplant recipients who retained their native kidneys, and with clinical observations made in normal-glucose-tolerant individuals with familial renal glucosuria in whom normal fasting plasma glucose levels were maintained despite markedly elevated rates of urinary glucose excretion [13, 14].

Following dapagliflozin administration, EGP declined in both the nephrectomy and residual kidney groups, but the decrement in EGP was greater in the bilateral nephrectomy group. The difference between the decline in EGP following dapagliflozin vs placebo treatment (1.11 ± 0.72 μmol min^−1^ kg^−1^) was significantly lower (*p* = 0.03) than that observed in renal transplant patients with both residual native kidneys. A number of mechanisms could explain these observations: (1) activation of the renal nerves transmit a signal to the liver to increase hepatic glucose production and complete renal denervation (i.e. transplanted kidney and removal of both native kidneys) results in loss of the neural signal; (2) the native but not the transplanted kidney releases a metabolite(s) and/or hormone(s) into the circulation that travels to the liver and stimulates the hepatic glucose production; (3) the renal nerves exert a local effect on the kidney to stimulate renal glucose production; or (4) some combination of these, or yet to be defined mechanisms.

To gain insight into the neuroendocrine response to SGLT2 inhibition, we measured the plasma concentrations of adrenaline and noradrenaline, being aware that the majority of released noradrenaline is taken up again by the nerve terminals and accurate assessment of noradrenaline turnover requires tracers [15]. Baseline plasma adrenaline levels were not significantly different in the two groups and did not change following dapagliflozin or placebo. Plasma noradrenaline concentration was similar at baseline in both groups and increased slightly in the residual native kidneys group after dapagliflozin while it decreased in the nephrectomy group (Fig. 6). A dedicated study using ^3^H-labelled noradrenaline would be required to examine whether the central nervous system plays any role in the dapagliflozin-induced stimulation of EGP.

A greater increase in urinary glucose excretion following dapagliflozin administration was observed in the group with residual native kidneys compared with the bilateral nephrectomy group. This difference cannot be explained by the differences in GFR, since this was similar in both groups. It is possible that in individuals with residual native kidneys, tubular reabsorption of glucose was impaired even though the residual native kidneys contributed significantly to the GFR [16, 17]. Further, kidney innervation may play an important role in the expression and/or function of SGLT2 [18, 19].

In the present study we observed a significant correlation between the amount of glucose lost in the urine and the stimulation of EGP following dapagliflozin administration in renal transplant patients with or without residual native kidneys. This novel physiological response is essential in order to prevent hypoglycaemia and maintain the fasting plasma glucose concentration constant at the basal level (Fig. 1), although it remains to be determined whether glucose handling in the kidney is the trigger for such a signal. Osmotic diuresis caused by SGLT2 inhibition can account for a rapid plasma volume contraction [20]. A 5% reduction in plasma volume can then activate the sympathetic nervous system which, in turn, could activate liver gluconeogenesis. Irrespective of the triggering signal, the rapidity of the response also suggests the presence of a neural signal. Since neither the plasma glucagon or insulin/C-peptide concentrations nor the glucagon:insulin ratio changed after administration of dapagliflozin in either group of transplanted individuals, it is unlikely that these hormones are involved in the SGLT2 inhibitor-induced stimulation of EGP. Following the initial report of Bonner et al [21] claiming a direct stimulatory effect of SGLT2 inhibitors on the alpha cell, recent studies in a more physiological setting showed that increased plasma glucagon during SGLT2 inhibitor treatment is unlikely to result from direct inhibition of SGLT2 in alpha cells [22]. Despite the fact that co-administration of a glucagon like peptide-1 receptor agonist (GLP-1RA) prevents SGLT2 inhibitor-mediated increases in plasma glucagon and decreases in plasma insulin, EGP increases persisted, making it unlikely that EGP increases are the consequence of increased glucagon:insulin ratios [23].

Following dapagliflozin administration, plasma βOHB levels increased progressively and significantly, more pronouncedly in individuals with residual native kidneys (Fig. 5). The increase in plasma βOHB concentration cannot be explained by changes in plasma insulin or glucagon concentrations or by altered plasma NEFA levels. Surprisingly, the change in βOHB levels occurred in the absence of changes in the rate of substrate oxidation [24, 25]. Whether the higher levels of noradrenaline in nephrectomised individuals contributed to the increase in βOHB concentrations remains to be determined.

We and others [2, 24] have reported previously that the decline in plasma glucose concentration following dapagliflozin treatment is associated with a shift of energy metabolism from glucose to fat oxidation in individuals with type 2 diabetes. In the present study we did not observe any change in substrate oxidation rates, consistent with findings from our previous studies in non-diabetic individuals [26]. This is not surprising because in individuals with normal glucose tolerance, dapagliflozin-induced glucosuria is not associated with any change in circulating substrate (glucose, NEFA) or hormone (insulin, glucagon) concentrations.

Potential limitations of the present study include its short duration, making extrapolation of results to chronic dapagliflozin administration tenuous. The number of participants is relatively small but the study population was carefully selected to include only individuals with autosomal dominant polycystic kidney disease to ensure a homogeneous group of participants [27]. Further, it is difficult to find large numbers of participants who undergo bilateral nephrectomy prior to kidney transplantation. In addition, the chronic use of prednisone should be considered as this may have had an effect in sustaining basal EGP, though a similar stable dose was used in all study participants.

In summary, by studying nephrectomised transplant recipients we have identified a unique opportunity to uncover a fundamental aspect of physiology that regulates the renal–hepatic axis.

The present results implicate the existence of a novel neuronal axis linking the kidney and the liver that modulates EGP in response to the glucosuria induced by SGLT2 inhibition. We hypothesise that SGLT2 inhibition activates efferent renal nerves, generating a signal that augments endogenous (hepatic) glucose production, where the stimulus is elaborated to elicit a sympathetic stimulation to sustain glucose production to compensate for the urinary glucose loss and, thus, prevent hypoglycaemia. In keeping with our hypothesis, persistence of native kidneys (i.e. preserved kidney innervation) was associated with a full compensatory EGP activation, while kidney denervation (i.e. individuals with kidney transplantation with no residual native kidneys) was associated with a blunted compensatory EGP increase.

## Electronic supplementary material

ESM 1 (PDF 490 kb)

## Data Availability

The datasets generated during and/or analysed during the current study are available from the corresponding author on reasonable request.

## Abbreviations

EGP: Endogenous glucose production
βOHB: β-Hydroxybutyrate
*R*_d_: Rate of disappearance
SGLT2: Sodium–glucose cotransporter 2
TTR: Tracer:tracee ratio
