# Supplementary material for: Increase in endogenous glucose production with SGLT2 inhibition is attenuated in individuals who underwent kidney transplantation and bilateral native nephrectomy
Source: Diabetologia. 2020 Aug 22;63(11):2423–33. doi: 10.1007/s00125-020-05254-w (PMC7527374; doi:10.1007/s00125-020-05254-w)
Supplement: Supplementary file 1 — (PDF 490 kb) [file 125_2020_5254_MOESM1_ESM.pdf]

Electronic Supplemental Material

	**Residual Native Kidneys (n=10)**				**Bilateral nephrectomy (n=10)**			
	PLACEBO		DAPA		PLACEBO		DAPA	
	Baseline	After	Baseline	After	Baseline	After	Baseline	After
Glucose OX#	0.60±0.17	0.59±0.26	0.48±0.14	0.63±0.21	0.68 ±0.24	0.58±0.16	0.41±0.11	0.59±0.22
Lipid OX#	2.37±0.25	2.28±0.17	2.54±0.20	2.45±0.19	2.81±0.22	2.73±0.29	2.66 ±.08	2.61±0.13
Protein OX#	2.08±0.16	2.05±0.26	1.96±0.21	1.92±0.31	1.75±0.19	1.70±0.17	1.54 ±0.18	1.59±0.20
NOGD#	3.94±0.82	3.34±0.48	4.52±0.47	4.15±0.55	4.83±0.38	4.74±0.53	4.86±0.31	4.53±0.42
REE (kJ/day)	6598±493	6665±359	6702±351	6711±217	6522±175	6443±297	6204±322	6322±301

ESM Table 1: Indirect calorimetry parameters at baseline and in response to placebo and dapa in both groups. OX, oxidation; NOGD, non-oxidative glucose disposal; # mg/KgFFM/min; REE, rate of energy expenditure. FFM: free fat mass

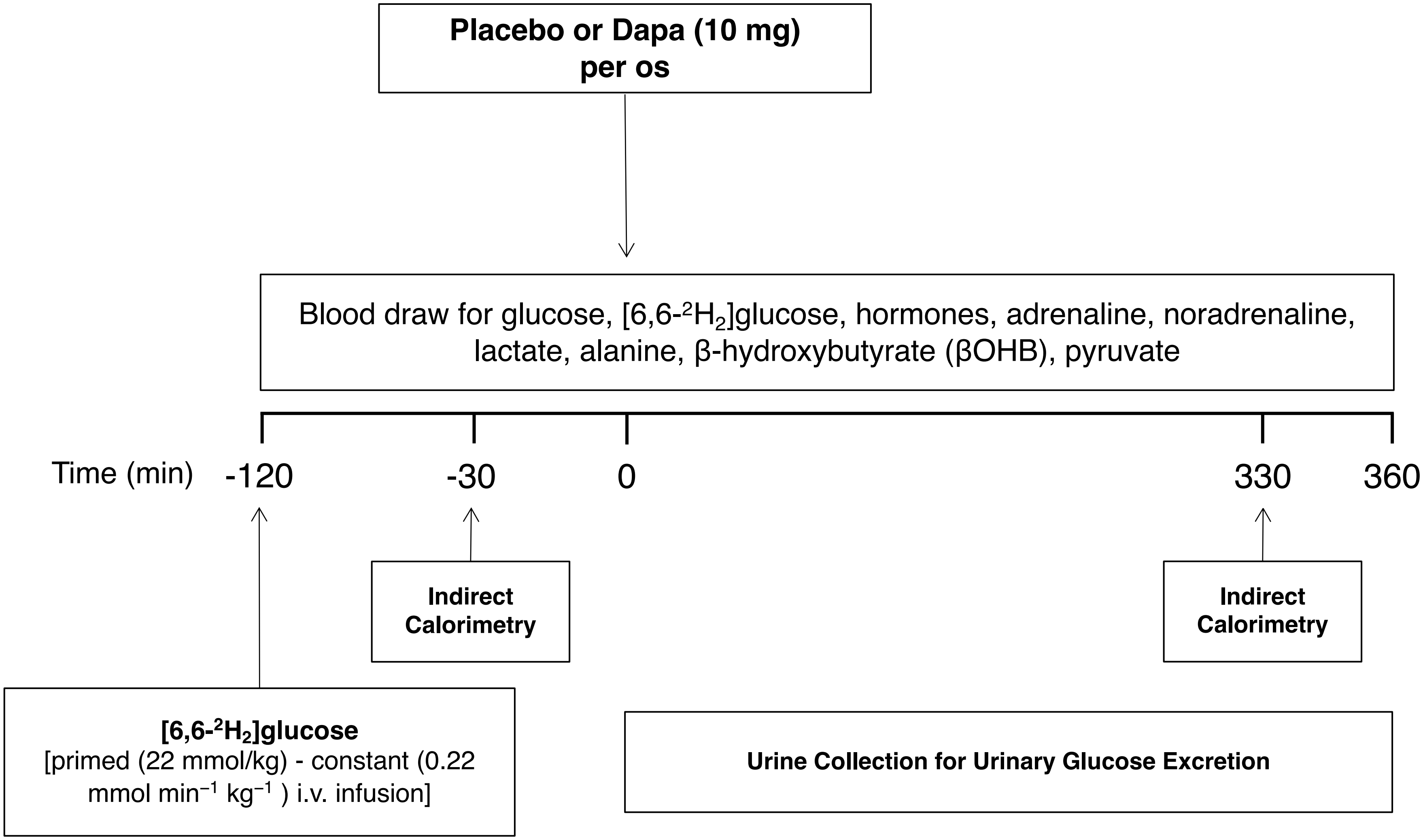

ESM Fig. 1: Detailed experimental procedures

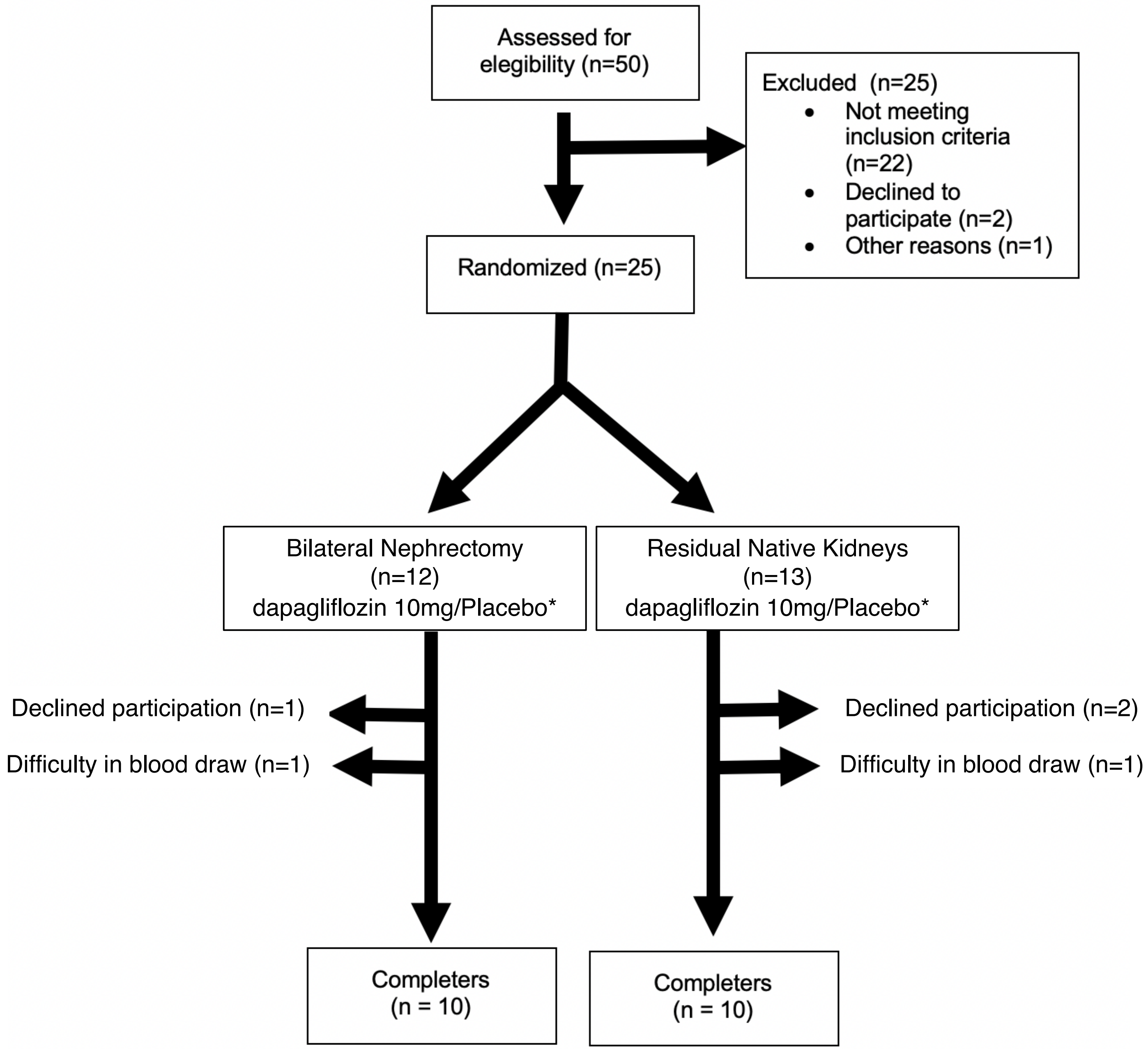

ESM Fig.2: Study Design and Patient's disposition. *Both groups underwent two identical experimental procedures with administration of a single dose of dapagliflozin 10 mg and placebo at 5-14 day interval in a random order.
